# Paralleling insulated-gate bipolar transistors in the H-bridge structure to reduce current stress

**DOI:** 10.1007/s42452-021-04420-y

**Published:** 2021-03-02

**Authors:** Majid Memarian Sorkhabi, Karen Wendt, Daniel Rogers, Timothy Denison

**Affiliations:** 1 MRC Brain Network Dynamics Unit, University of Oxford, Oxford, OX1 3TH UK; 2 Department of Engineering Science, University of Oxford, Oxford, OX1 3PJ UK

**Keywords:** Transcranial magnetic stimulation, Pulse generator, Parallel IGBTs, Flexible TMS pulse, Current stress

## Abstract

In this study we present the new power electronic circuit implementation to create the arbitrary near-rectangular electromagnetic pulse. To this end, we develop a parallel- Insulated-gate bipolar transistors (IGBT)-based magnetic pulse generator utilizing the H-bridge architecture. This approach effectively reduces the current stress on the power switches while maintaining a simple structure using a single DC source and energy storage capacitor. Experimental results from the circuit characterization show that the proposed circuit is capable of repeatedly generating near-rectangular magnetic pulses and enables the generation of configurable and stable magnetic pulses without causing excessive device stresses. The introduced device enables the production of near-rectangular pulse trains for modulated magnetic stimuli. The maximum positive pulse width in the proposed neurostimulator is up to 600 µs, which is adjustable by the operator at the step resolution of 10 µs. The maximum transferred energy to the treatment coil was measured to be 100.4 J. The proposed transcranial magnetic stimulator (TMS) device enables more flexible magnetic stimulus shaping by H-bridge architecture and parallel IGBTs, which can effectively mitigate the current stress on power switches for repetitive treatment protocols.

**Supplementary Information:**

The online version contains supplementary material available at 10.1007/s42452-021-04420-y.

## Introduction

Transcranial magnetic stimulation (TMS) is a non-invasive technique that can activate cortical neurons using electromagnetically induced stimuli. TMS works by passing a transient current through a treatment coil placed on the patient's head, thus inducing an electric field which safely penetrates the skull. It has a long history of applications in both neuroscience research and clinical therapies [1, 2]; The US food and drug administration (FDA) approved TMS for the treatment of several psychiatric and neurological diseases, such as major depressive disorder and obsessive–compulsive disorder (OCD) and it is under investigation for many other therapies [1, 3]. Delivering TMS stimuli in a long sequence of pulses (trains or bursts) is called repetitive TMS (rTMS) which has been widely utilized in non-invasive treatments for several neurodegenerative diseases [4, 5]. Repetitive neuromodulation protocols can induce long-term neuroplastic changes in brain circuits [6]. In therapies called theta-burst stimulation (TBS), the repetition of pulses reaches 50 Hz [7], while this rate can reach 666 Hz in Quadri-pulse stimulation (QPS) protocol, with longer time intervals between trains (five seconds) and fewer pulse repetitions per train (four pulses) [8]. QPS method uses four monophasic stimuli and can induce a significant aftereffect on cortical areas [9].

**Fig. 1:**
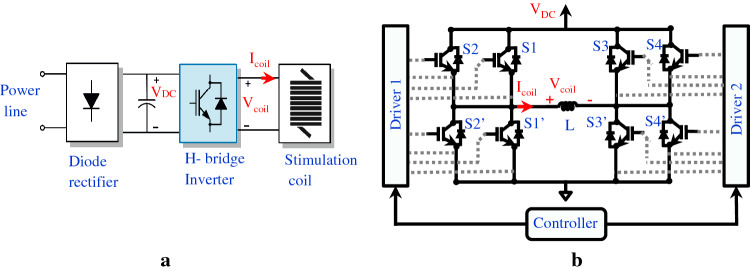
The proposed structure for the TMS device. **a** The overall diagram of the implemented system. **b** Parallel-IGBTs architecture for the inverter block to reduce the current stress in the H-bridge

The operating principle of a conventional TMS pulse generator is simple: a large energy storage capacitor (C $\approx$ 250 µF) is charged to a DC voltage of about 1.6 kV(maximum). When the power switch (generally a thyristor) is gated into conducting state, the pre-charged capacitor is discharged through a treatment coil (L) and generates a fast-changing field. The coil inductance is between 15 and 24 µH and the maximum current flowing in the stimulation coil is 5 kA (peak-to-peak 10 kA). The maximum magnetic field produced at the coil surface can reach 1 T in average. The basic structure of this circuit is shown in Fig. 1a. Despite the success of the TMS method, there are some vital limitations associated with the pulse shape parameters. Due to the structure of the LC resonant circuit in the available pulse generators, the waveforms produced in these devices are fixed and dependent on hardware parameters. Thus, the magnetic stimulus is generally cosine-shaped with a period of 400 microseconds (a so-called ‘biphasic pulse’) [10]. More adjustable control of the stimulus waveform could potentially enable novel research and clinical applications that are not achievable with conventional TMS equipment [11].

Addressing this need, Gattinger et al. introduced new TMS device, called ‘flexTMS’ [12]. This device utilized one DC-link source and a H-bridge structure to manage the LC resonance at different time intervals. To enhance magnetic stimulation flexibility, Peterchev et al. have designed a controllable TMS (cTMS) device to generate flexible near-rectangular pulse shapes [13]. Four insulated-gate bipolar transistor (IGBT) switches incorporating freewheeling diodes, which form the two half-bridges architecture, were utilized to connect the stimulation coil to the energy storage capacitors, as shown in Fig. 1sb. Two isolated DC sources and separate energy storage capacitors (C_1_, C_2_) have been used and the output pulse can produce four different voltages, V_coil_ = {V_DC1_, − V_DC2_, V_DC1_ − V_DC2_, 0}. Although deploying two separate capacitors can increase the output voltage level, recharging the capacitors is a challenging task when returning energy from the coil (regeneration mode).

Another limitation in the particular implementation of the cTMS device reported in [13] is the current overload imposed on the switches (the peak current was shown to be up to 2.5 times the nominal IGBT values). In power-electronic systems, power semiconductor elements are one of the most fragile components [14]. Reviews of the effect of overcurrent on the IGBT switches are provided by [15, 16]. As a result of current overload, physical signatures (such as discoloured spots on the surface) are observed at the IGBT die-level. Although these signatures do not necessarily cause immediate failure of the device, they have been observed to significantly decrease the lifetime of the device and increase the risk of sudden failure [17]. The importance of overcurrent rating of devices is critical in protocols such as rTMS, which require very high peak currents (but at a very low duty-cycle). Such usage is more likely to cause ‘invisible’ accumulating damage in the semiconductor devices (leading to eventual failure), when compared to the simpler steady-state heating process that is observed in continuous duty-cycle applications. This is particularly important in a medical equipment application, such as a TMS machine, where the safety of the patient and operator may be at risk.

This research consists of the following sections: in the first section, the proposed structure for the magnetic stimulator device is introduced. Then the driver design for the equal current distribution between parallel-IGBTs is explained. The measured results of the experimental prototype are given in the results section. Finally, the discussion about the key findings, limitation of the proposed circuit and conclusion are presented.

## Materials and methods

**Fig. 2:**
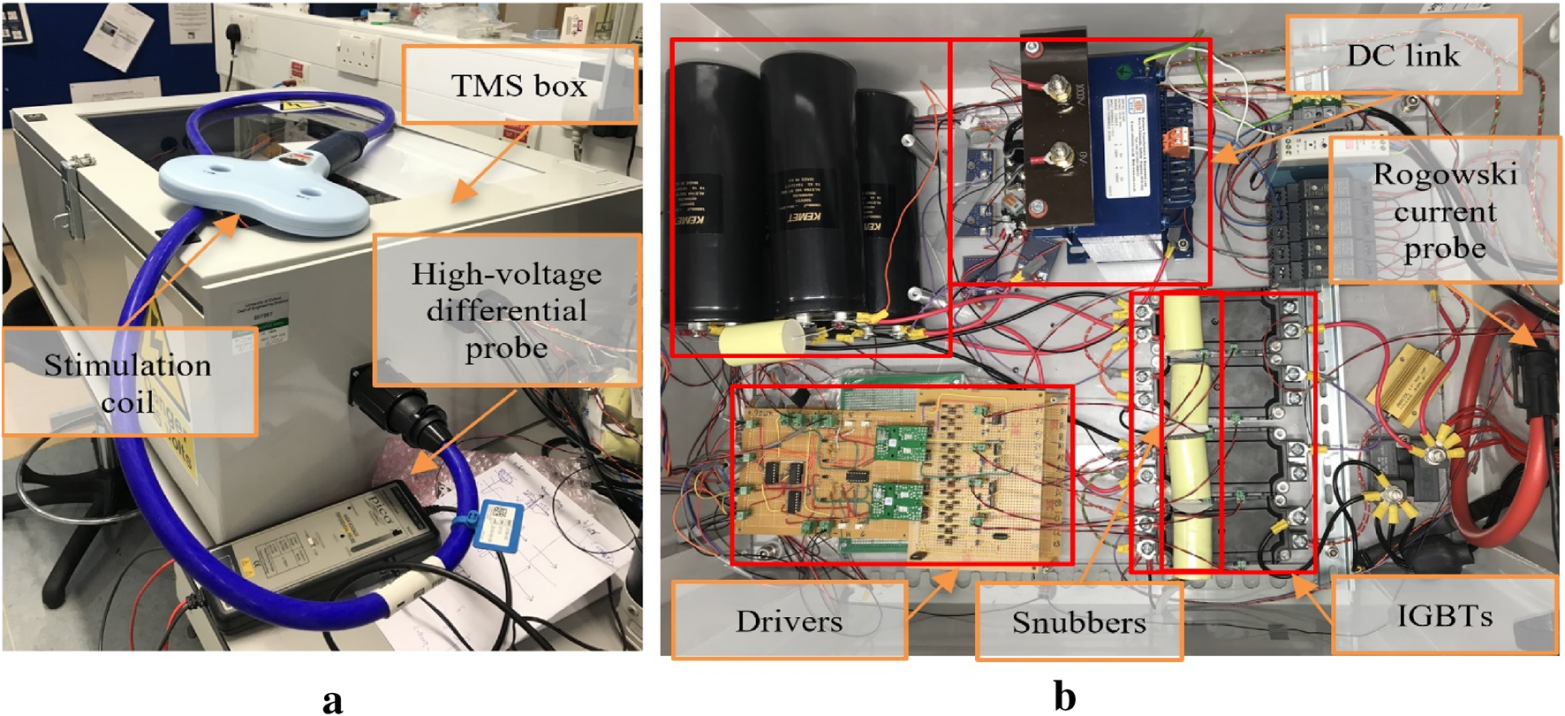
Experimental TMS Setup. **a** Physical assembly and stimulation coil. **b** Internal hardware for the TMS box. The coil voltage and current were measured via a high-voltage differential probe (TA044, PICO TECHNOLOGY, UK) and a Rogowski current probe (I6000S FLEX-24, FLUKE, USA), respectively

As illustrated in Fig. 1a, the mains ac voltage is converted to the DC voltage by full-wave diode rectifier, then DC-link capacitors are charged (V_DC_). Then the H-bridge inverter generates the near-rectangular pulse waveform from the DC voltage with frequency switching concept. Depending on the required DC voltage level, a step-up transformer may be placed before the rectifier. The proposed circuit uses eight IGBT switches to reduce the current stress, forming an H-bridge, as shown in Fig. 1b. The complete laboratory TMS setup is represented in Fig. 2 which are connected to the stimulation coil (L).

The proposed device is controlled by a MicroLabBox (dSPACE GmbH, Germany) digital control system (controller). IGBTs are connected in parallel in order to increase the current capability. The H-bridge structure is advantageous when compared to the cTMS structure as it requires only one DC source. However, some disadvantages of the H-bridge structure must be considered. The manufacturing process variations can cause tolerances in power switch parameters such as stray inductance. In addition to them, the parasitic inductance of the power circuit and different propagation delays in the driver systems may increase asymmetrical current sharing between parallel-connected IGBT switches [18].

The driver circuit plays a key role in resolving these problems. For instance, in the individual driver concept, where each IGBT module has a separate driver, differences in the signal propagation times to the IGBTs, dissimilar gate-emitter voltages, and jitter (time offset due to the digital drivers having their own system clock) are the main factors for the asymmetric distribution of current. In contrast, in the central driver concept, all the parallel switches are controlled by one driver. As long as the single driver is suitably sized so that it can provide the necessary current to all the IGBTs connected to it, the turning-on and turning-off speed of the switches is not reduced.

In power circuits, if high currents are switched rapidly, the stray inductance in the circuit causes a voltage overshoot. This overvoltage may overstep the maximum blocking voltage of the IGBTs that can damage the power switches [19]. The snubbers can effectively protect against voltage overshoot during the switching transitions and keep an IGBT in the safe operation area. Snubbers are connected in parallel to the Emitter–Collector of IGBTs, as shown in Fig. 2 The DC link contains a full-wave diode rectifier, a capacitive charge limiting resistor with four pulse capacitors. This block rectifies the voltage received from the main socket and charges the capacitors. The capacitor series structure increases its operating voltage (up to 1000 V), and its parallel structure increases capacitance (C_tot_ = 10 mF).

## Driver

**Fig. 3:**
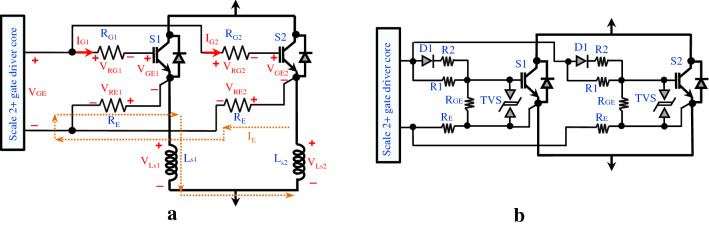
The proposed central driver concept for two parallel IGBTs: **a** Balancing effect of the emitter resistors. **b** Proposed gate driver interface of two parallel IGBTs and Scale 2 + gate driver core

In this research, the central driver is selected and implemented, as demonstrated in Figs. 1 and 3. As shown in Fig. 3a, suppose two parallel IGBTs (S1 and S2) are connected to the driver directly and there is no resistance between the emitters and the driver (R_E_ = 0). Dissimilar stray inductances (L_s1_ ≠ L_s2_) or different switching behaviour result in different voltage drops (V_Ls1_ ≠ V_Ls2_). This voltage difference will cause static and dynamic imbalances between the two emitter currents (I_E1_, I_E2_).

Current sharing between IGBTs can be encouraged by adding two low-ohmic-value resistors between the emitters and the driver (R_E_): if one IGBT turns on more quickly, its rate-of-rise of collector current will be greater, and so a greater voltage drop will be created across its emitter inductance, this in turn reduces the effective Gate-Emitter voltage seen by the IGBT, causing its turn-on speed to be reduced. Under the assumption that $\left( {I_{C1} + I_{C2} } \right) \gg I_{E}$ this can be modelled by

1 $${\Delta }V_{E} = V_{E1} - V_{E2} = \frac{{{\text{d}}I_{C1} }}{{{\text{d}}t}}L_{s1} - \frac{{{\text{d}}I_{C2} }}{{{\text{d}}t}}L_{s2}$$

2 $$I_{E} = \frac{{{\Delta }V_{E} }}{{2R_{E} }}$$

3 $$V_{GE1} = V_{GE} - I_{G1} R_{G1} - R_{E} I_{E} = V_{GE} - \frac{{{\Delta }V}}{2}$$

4 $$V_{GE2} = V_{GE} - I_{G2} R_{G2} + R_{E} I_{E} = V_{GE} + \frac{{{\Delta }V}}{2}$$

It can be seen that the faster switching device (higher di/dt) experiences a reduction in gate-emitter voltage (which will tend to decrease its switching speed) and the slower switching device (lower di/dt) will experience an increase in gate-emitter voltage (which will tend to increase its switching speed)—i.e. a negative feedback system has been formed [20].

In essence, the emitter resistors allow unequal gate-emitter voltages for the two IGBTs by limiting the circulating current I_E_ to a reasonable value. The choice of emitter resistor value should be high enough such that the magnitude of I_E_ is suitably limited, but low enough to ensure the Gate-Emitter capacitance can be charged/discharged by the gate drive suitably quickly. If the value is high, the switching speed of both IGBTs will be reduced (causing power losses to increase). If the value of resistance is increased too far, transient instability may result. In practice, a choice of resistor value that causes a peak circulating current I_E_ equal to the driver rating is reasonable (in this application, $I_{E} \approx 20 {\text{A}}$, compared to $I_{C} \approx$ 1.8 kA).

Artificially increasing the emitter inductances $L_{s1}$ and $L_{s2}$ beyond that naturally occurring in the circuit will tend to increase the strength of the balancing effect. Such an increase should be made with caution as it will tend to decrease stability and may led to destructive oscillatory or double-switching behaviour in the IGBTs.

Note that in each leg, the upper and lower switches are complementarily controlled and under no circumstances should they be turned on at the same time. This will short-circuit the DC link (termed a ‘shoot-through condition’) and likely destroy the circuit. Such behaviour can occur due to asymmetric delay times of control signal paths. Dead-time circuits may be used to prevent this [21].

The high switching speeds of IGBTs are an intrinsic source of electromagnetic interference (EMI) [22]. These EMI sources are readily coupled to nearby cables and printed circuit boards. Remedial actions (such as adding shielding) may be necessary to prevent undesired behaviour. Finally, to protect the sensitive gate-emitter terminal from voltage transients induced by driver output, EMI and other temporary voltage events, transient voltage suppression diodes (TVS) can be incorporated into the gate driver circuit. The final structure of the driver is illustrated in Fig. 3b and proposed neurostimulator components in Table 1. The gate driver is a two-channel driver core that provides a voltage swing of + 15 V/–8 V. The application of negative off-state voltage helps prevent the IGBTs from being turned on unintentionally. It is estimated that the overall parasitic inductance is $L_{S} \approx 120{\text{ nH}}$ for each IGBT. The optimal R_E_ is found by increasing the value, starting at 0.1 Ω, until satisfactory current sharing is achieved (within 5%).

**Table 1:** Key components of the proposed magnetic pulse generator

Component	Assignment	Rating	Part Number	Manufacturer
S1-S4 and S1′-S4’	IGBT	1.2 kV-1.8 kA	SEMiX603GB12E4p	Semikron
L	Stimulation coil	15.5 µH	D70 Remote Coil	Magstim
Scale 2 + driver core	Gate driver core	VGE_on_ = 15 V, VGE_off_ = −8 V	2SC0106T2A1-12	Power Integrations
DC-link capacitor	Pulse capacitor	10,000 μF, 500 VDC	ALS70A103NT500	KEMET Electronics
TVS	Transient V. suppressor	V_Break-down_ = ± 19.7 V	SMBJ16CA	Littelfuse Inc
R1, R2	Turn off and turn on resistor	22 Ω	RCC025 22R J	Arcol
R_GE_	Gate-emitter resistor	22 kΩ	RCC050 22 K J	Arcol
R_E_	Feedback resistor	500 mΩ	AP821 R5 J	Arcol
Controller	Digital controller	Time res.: 10 ns	MicroLabBox	dSPACE

## Results

**Fig. 4:**
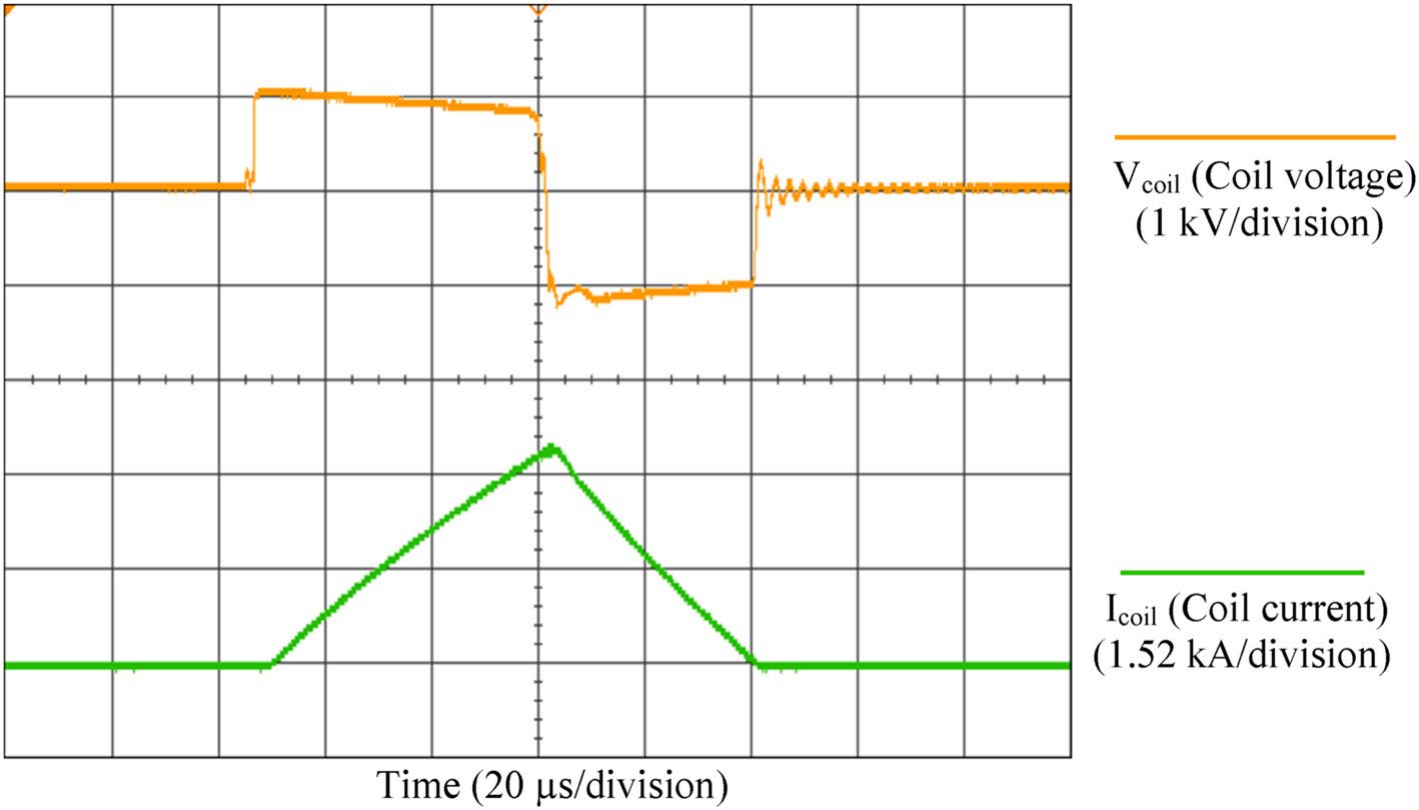
Measured coil voltage (V_coil_) and coil current (I_coil_) for 95 µs square stimulus (55 µs positive and 40 µs negative phase). The measurement locations of (V_coil_) and (I_coil_) parameters are shown in Fig. 1

The magnetic pulse generator has been characterized experimentally. For instance, a 95 µs square stimulus (55 µs positive and 40 µs negative phase) is shown in Fig. 4. The values of coils’ voltage and current are dependent on the coil inductance, the stimulus pulse width and the amplitude of the capacitor voltage. The resulting output pulse has three different voltage levels, V_coil_ = {V_DC_, − V_DC_, 0}; V_DC_ is adjustable via the variable autotransformer. The circuit was investigated with a DC-link voltage of V_DC_ = 1000 V and a peak output current of up to 3.6 kA (peak-to-peak coil current 7.2 kA). The maximum positive pulse width in the proposed TMS device is up to 600 µs, which is adjustable by the operator at a step resolution of 10 µs. The maximum transferred energy to the treatment coil was measured to be 100.4 J. The measurements were done with a digital oscilloscope and 250 MSa/s sampling rate, without any switching spikes filtering.

**Fig. 5:**
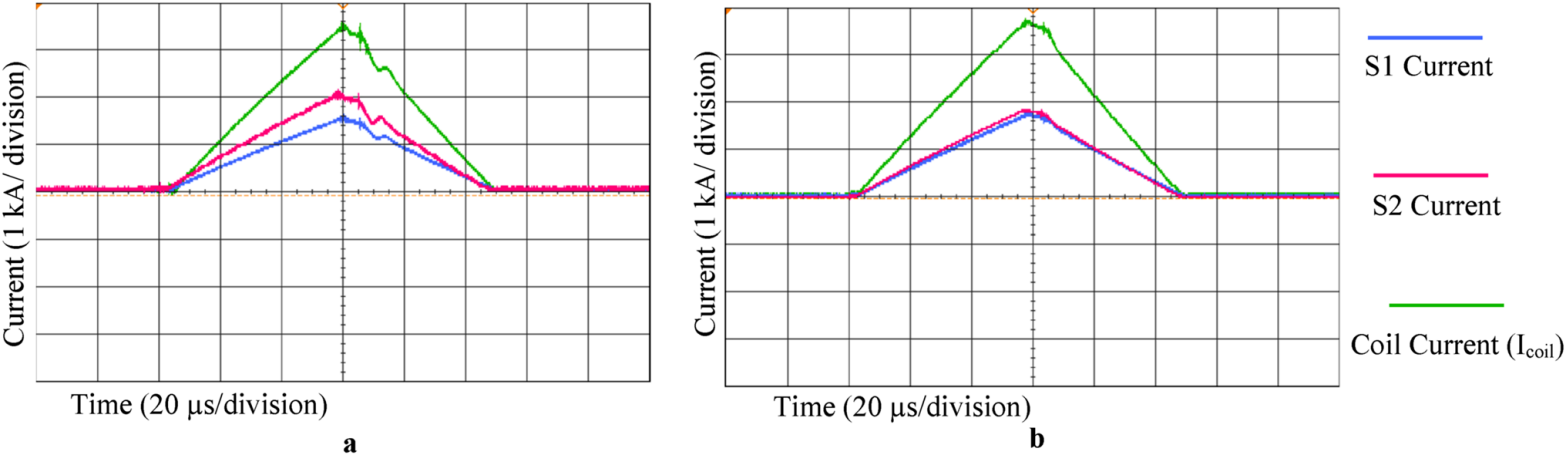
Effect of Emitter feedback on IGBT current sharing; **a** Absence of emitter resistors; **b** Emitter resistors included. It is seen that the lack of feedback in the Emitter loop causes an unequal distribution of current in the IGBTs. Each square represents 1 kA current in 20 µs

To investigate the effect of the presence of the Emitter resistances on the parallel-IGBTs current balance, two experiments were performed in two different states. For this purpose, a stimulus waveform similar to Fig. 4 was produced with and without emitter resistor. The currents were measured in the parallel switches S1 and S2, according to the circuit in Fig. 3b, and the peak coil current was I_coil_ = 3.2 kA. In the first experiment, the Emitter resistances were set to zero (R_E_ = 0). The results of the current measurements of each IGBT are shown in Fig. 5a. As a result of the different gate-emitter voltages in S1 and S2, arising from the different voltage drops in the parasitic inductors, the current sharing is not equal. The current of S1 and S2 is 40 and 60% of the total output current, respectively. By adding Emitter resistances and repeating the experiment (R_E_ = 0.5 Ω, according to Table 1), the results of Fig. 5b are obtained. The currents are almost symmetrically divided. Therefore, it can be concluded that the proposed structure for two parallel switch drivers was able to achieve an acceptable current sharing balance.

## Discussion

The implemented TMS circuit is based on the new paradigm that proposed to use the H-bridge and parallel- IGBTs technique that can change the magnetic stimulus waveform and reduce current stress on the IGBTs. Current magnetic stimulators restrict the implementation of new magnetic stimulation protocols in the TMS tests. Most of these restrictions are due to the circuit principles by which magnetic stimulators work. One of the key technical limitations of them is the pulse shape and pattern which can restrict the clinical effectiveness of the TMS devices and limit their potential in research. Also producing sequential and stable stimuli at high repetition rates is one of the main challenges of this method. The output of the proposed device develops beyond conventional stimuli such as rectangular or damped Cosine waveforms and proceed toward an arbitrary stimulus. Novel stimuli shapes may have practical benefits over the pulses produced by current-generation magnetic stimulators for clinical trials. Since parallel IGBTs allow the generation of near-rectangular stimuli with a high repetition rate, the pulse train can be used to generate magnetic stimuli with modulation techniques, such as pulse width modulation (PWM). The modulation method enables the management of the output waveform, frequency, and pattern of the treatment paradigm with cost-effective and reliable methods. More details on the modulation approach in the TMS devices are available in [23].

**Fig. 6:**
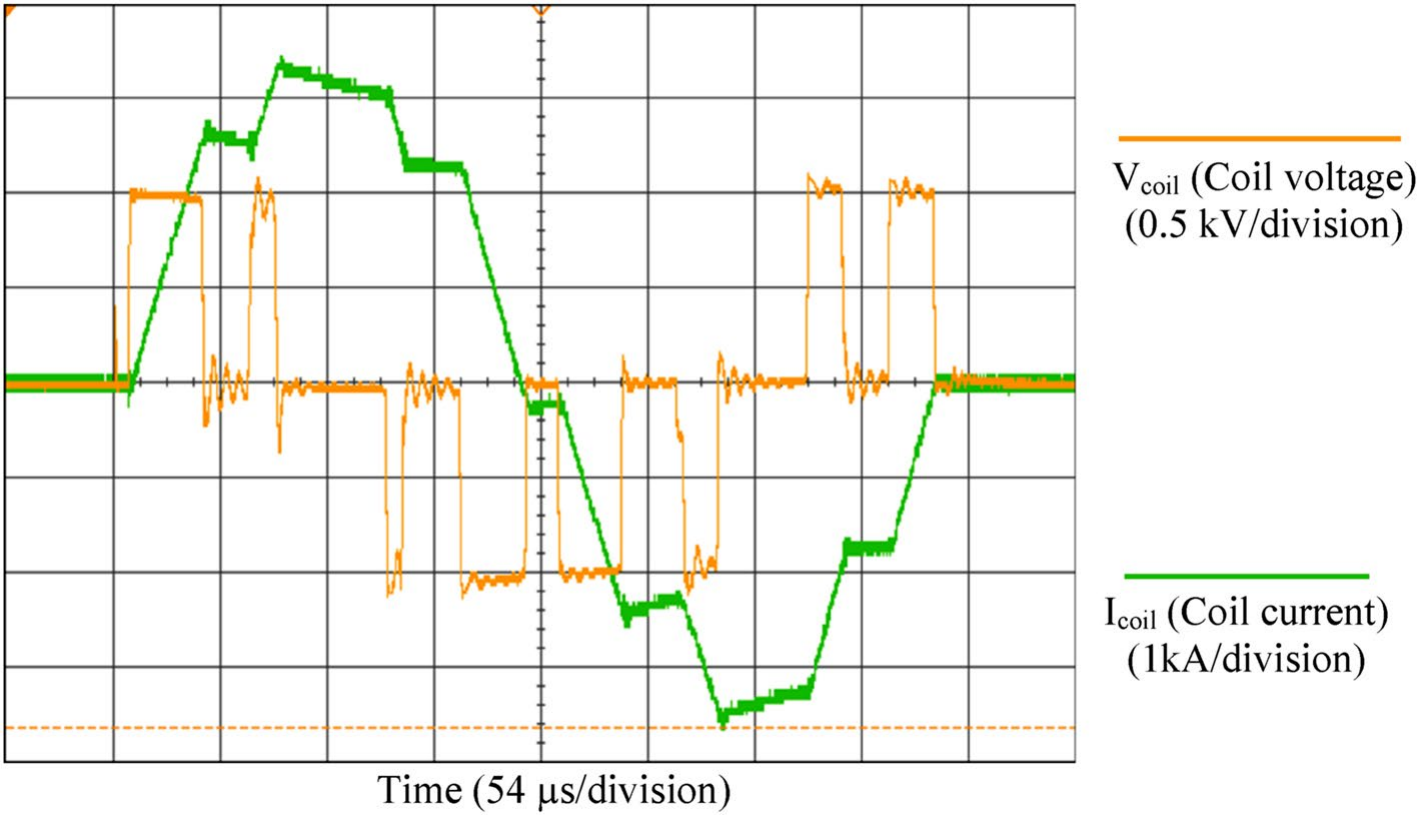
Measured waveforms for 2.5 kHz biphasic stimulus (PWM-equivalent for the Cosine stimulus)

Figure 6 is an example of generated PWM magnetic stimuli to mimic a 2.5 kHz cosine pulse. The intrinsic low-frequency nature of the neural tissues attenuates the high-frequency harmonics of the pulse, and the membrane voltage changes will be close to ideal [23].

## Conclusion

TMS devices play a fundamental role in many non-invasive brain modulation solutions in various fields of diagnostics and clinical neuroscience. The proposed neurostimulator enables more flexible magnetic stimulus shaping by H-bridge architecture and parallel IGBTs. As well, the controllable stimulus shaping can potentially enhance the neural population selectivity [24]. One of the major concerns in the new designs of TMS devices is the large current stress applied to the power switches and the associated risks of device failure. Paralleling IGBTs becomes necessary for TMS devices with higher output power ratings and duty cycles (such as high frequency rTMS or QPS) where a single IGBT is not adequate. Parallel IGBTs introduce challenges in keeping an equal current distribution while ensuring a fast turn-on and turn-off time; the central driver concept and IGBT Emitter resistors can be used to achieve this.

One of the limitations of the proposed design is the relatively large size of the implemented circuit, compared to conventional TMS systems. Also, due to the sequential switching of the IGBTs, there is a possibility of increasing switching power losses and the higher die temperature, which should be further investigated for repetitive TMS protocols. The effects of high-frequency harmonics, induced by the rectangular magnetic stimuli, on the neural behavior should also be further examined.

In summary, the necessity of using parallel IGBTs to decrease current stress and to maintain all switches within their safe operating area was investigated. The proposed circuits have been experimentally characterized. The measured results indicated that the proposed driver circuit can distribute equal current between parallel IGBTs, in both static and dynamic states, while safely generating pulses with a width of up to 600 μs.

## Supplementary Information

Below is the link to the electronic supplementary material.

Supplementary file1 (PDF 32 KB)
