# Supplementary material for: Paralleling insulated-gate bipolar transistors in the H-bridge structure to reduce current stress
Source: SN Appl Sci. 2021 Mar 2;3(4):406. doi: 10.1007/s42452-021-04420-y (PMC7925468; doi:10.1007/s42452-021-04420-y)
Supplement: Supplementary file 1 — Supplementary file1 (PDF 32 KB) [file 42452_2021_4420_MOESM1_ESM.pdf]

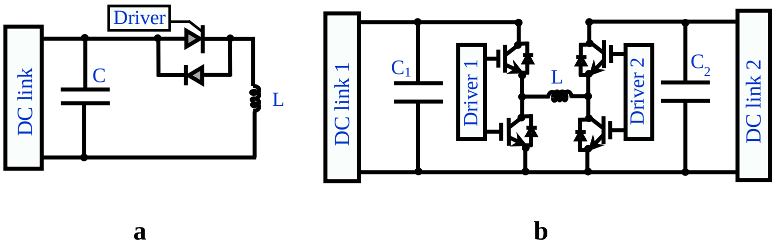

Fig. 1s Typical pulse generator architectures in TMS devices. a) LC resonant circuit. b) cTMS device structure.
